# Psychiatric Symptoms in Acute and Persisting Forms of COVID-19 Associated with Neural Autoantibodies

**DOI:** 10.3390/antib12030049

**Published:** 2023-07-27

**Authors:** Niels Hansen

**Affiliations:** Department of Psychiatry and Psychotherapy, University Medical Center Göttingen, Von-Siebold-Str. 5, 37075 Göttingen, Germany; niels.hansen@med.uni-goettingen.de

**Keywords:** neural autoantibodies, COVID-19, post-COVID syndrome, psychiatry, autoimmunity

## Abstract

(1) Background: In this narrative review, we focus on neural autoantibodies in patients with coronavirus disease 2019 (COVID-19) as a consequence of severe acute respiratory syndrome coronavirus type 2 infection and persisting symptoms of post-COVID-19 syndrome with a psychiatric presentation. (2) Methods: Our methods include using the PubMed database to search for appropriate articles. (3) Results: We first describe the phenomenon of the psychiatric manifestation of COVID-19 in acute and persistent forms, associated with neural autoantibodies, often attributable to encephalopathy or encephalitis. We discuss the spectrum of neural autoantibodies in neuropsychiatric patients affected by COVID-19 and post-COVID-19 syndrome. Evidence from our research suggests that it is highly likely that neural autoantibody production is facilitated by SARS-CoV-2 infection, and that more neuropsychiatric patients than control subjects will present neural autoantibodies. (4) Conclusions: These observations support the hypothesis that acute and persisting forms of COVID-19 promote autoimmune diseases. Our patients therefore require comprehensive evaluation to avoid overlooking such autoantibody-associated psychiatric disorders associated with COVID-19.

## 1. Psychiatric Disease Manifestation in COVID-19 Associated with Neural Autoantibodies

Neural autoantibodies are commonly reported in neuropsychiatric patients diagnosed with coronavirus 2019 (COVID-19) [1,2,3,4] secondary to infection with severe acute respiratory syndrome coronavirus type 2 (SARS-CoV-2). Less is known about acute and persisting forms of COVID-19 with neural autoantibodies [5] and predominant psychiatric manifestation. Therefore, we describe the phenomenon of psychiatric symptoms in COVID-19 associated with neural autoantibodies in this review. The spectrum of neural autoantibodies in neuropsychiatric patients affected by COVID-19 and post-COVID-19 syndrome is presented. Evidence from studies suggests that neural autoantibody production is most likely favored by SARS-CoV-2 infection [3,6], and more patients with neuropsychiatric symptoms have neural autoantibodies than control subjects [1,5]. These observations support the hypothesis that acute and persistent forms of COVID-19 promote autoantibody-associated brain disease with a psychiatric manifestation. Psychiatric patients in psychiatric institutions must therefore be comprehensively screened to avoid overlooking such autoantibody-associated psychiatric disorders associated with COVID-19.

## 2. Methodological Approach

In this narrative review article, PubMed was searched for the following keywords alone or in combination: autoantibodies, neural autoantibodies, neuronal autoantibodies, membrane surface autoantibodies, intracellular autoantibodies, COVID-19, long COVID-19, post-COVID-19 syndrome, psychiatry, psychiatric symptoms, and SARS-CoV-2. Articles were selected that, according to the author’s subjective evaluation, provided relevant research on the topic of the occurrence of neural autoantibodies in COVID-19 and PCS.

## 3. Psychiatric Symptom Spectrum in COVID-19 with Neural Autoantibodies

Coronavirus disease 2019 (COVID-19) triggering severe acute respiratory syndrome coronavirus type 2 (SARS-CoV-2) infection can be fatal and is one of the top ten causes of death worldwide [7]. Its clinical features vary widely and can lead to psychiatric symptoms such as cognitive impairment in several areas [8] or psychosis [4] (Table 1). Psychiatric symptoms and COVID-19 influence each other, as patients with no psychiatric history carry a higher risk for developing a psychiatric disorder. Moreover, having a psychiatric disorder is a potential risk factor for COVID-19 [9]. Psychiatric symptoms resulting from COVID-19 in persons with no previous psychiatric illness may be attributable to an organic brain disease such as encephalopathy or encephalitis [10] or could even develop after COVID-19 via other factors once the organic cause has been resolved. Other evidence from a longitudinal COVID-19 study [11] indicates that resilience increases over time, with little change in psychiatric symptoms. Neural autoantibodies may accompany psychiatric disorders [12] and psychiatric autoimmune encephalitis [13] and may be associated with encephalopathy. It is generally assumed that COVID-19 can trigger autoimmune processes involving the production of autoantibodies [3,14]. In this review, we specifically focus on neural autoantibodies in acute (COVID-19) and long-term forms of COVID-19 (long COVID-19 and post-COVID-19 syndrome (PCS)) in patients with psychiatric symptoms to highlight the importance of autoantibody testing in such patients (Table 1). Long COVID-19 is characterized by persisting symptoms lasting 4–12 weeks after COVID-19. In comparison, PCS refers to a time interval of ongoing symptoms 12 weeks after COVID-19 disease. We address the different forms of psychiatric COVID-19 manifestations along a longitudinal timeline, but of most interest are psychiatric symptoms potentially caused by PCS, as this could constitute a differential diagnostic challenge. Many patients present with psychiatric symptoms that first appeared in the context of COVID-19 disease, and the question arises as to whether or not these symptoms are caused by PCS. If they are, biomarkers such as neural autoantibodies are of particular value to evaluate the organic basis of symptoms in such patients. In the next section, we discuss the main potential mechanisms involved in producing autoantibodies in patients with psychiatric symptoms and COVID-19, long COVID-19, or PCS.

## 4. Pathomechanisms of Neural Autoantibody Production in COVID-19

There are several distinct proposed pathomechanisms involved in the production of neural autoantibodies in COVID-19 and even PCS, as delineated below. Furthermore, mechanisms such as extrafollicular B-cell activation in conjunction with the production of autoantibody-secreting B-cells [15] and the involvement of Toll-like receptor 7 [16] are important key players in autoantibody production, which are described further in this review.

### 4.1. Molecular Mimicry and Autoantibodies

A recent study [17] described eight SARS-CoV-2-associated patients suffering from neuropsychiatric symptoms, i.e., working memory impairment and psychiatric symptoms originating from anti-N-methyl-D-aspartate receptor (NMDAR) encephalitis. The authors speculated that possible mimicry between the nonstructural SARS-CoV-2 proteins and epitopes of NMDAR subunits may be the main reason for the immune response against NMDAR. There is evidence of considerable similarity between the SARS-CoV-2 virus and the mammalian proteome [18,19]. Indeed, a variety of peptides with more than six amino acid sequences share sequences with SARS-CoV-2 and human CNS proteins [19]. It is highly probable that the immune system produces antibodies that react with human proteins and that may also access the brain if the blood–brain barrier is leaky, which could occur with COVID-19. Since the neural autoantibodies react with structures belonging to our own central nervous system structures, molecular mimicry may be responsible for the production of neural autoantibodies.

### 4.2. Hyperstimulation of the Immune System in COVID-19

Another crucial mechanism of neural autoantibody production in COVID-19 is the hyperstimulation of the immune system by COVID-19 [14,20]. The immune system’s hyperactivation is triggered by an excessive release of cytokines, the so-called “cytokine storm” in COVID-19 [21,22]. The overproduction of several inflammatory mediators such as interleukin 1β (IL-1β), interleukin-2R, interleukin 6 (IL-6) up to interleukin 10, interferon-γ, monocyte chemoattractant protein 1A, macrophage inflammatory protein 1A/B, tumor necrosis factor-α, and vascular endothelial growth factor; overactivated and exhausted T-cells; and a rise in autoantibodies characterize the immune system’s hyperstimulation in COVID-19 [20,23,24,25]. The immune system’s overstimulation alone can cause the production of autoantibodies. Other factors, such as genetic factors like HLA-DRB1, may also cause its overstimulation [14]. The loss of self-tolerance, as seen in COVID-19 patients with autoreactive CD4+ and CD8+ cells and autoantibodies [20,23,26], probably promotes the development of neural autoantibody-associated psychiatric disease. A hyperstimulated immune system further demonstrates its inadequate response as it has difficulty terminating the inflammation and returning to a normal homeostatic state [27]. This inability to return to normal may be due to a deficiency in adoptive COVID-19-specific T cells, which can induce immune reconstitution. The prior existence of autoantibodies in patients, such as those that neutralize type I interferons with COVID-19, may even lead to a high risk of patient morbidity [28], indicating that the existence of autoantibodies can influence the patient’s outcome.

### 4.3. Neutrophils Extracellular Traps and Autoantibodies

During neutrophil blood cell death, neutrophil extracellular traps are released (NETs) that can mediate the damaging effects of neutrophils in the immune defense against a destructive antigen such as SARS-CoV-2. The increase in NETs in COVID-19 was determined by the fact that NET markers were increased in COVID-19 patients, e.g., the levels of cell-free desoxyribonucleic acid (DNA), myeloperoxidase DNA, and citrullinated histone H3, and some of these markers, such as cell-free DNA, correlated with the absolute neutrophil count and acute phase proteins [29]. These observations support the clinical relevance of the findings reported above. NET production is triggered by hyperinflammation promoted by the aforementioned cytokine storm [30]. There are also pro-NET processes triggered by SARS-CoV-2, such as autoantibody production [29].

### 4.4. A More Permeable Blood–Brain Barrier and Autoantibodies

SARS-CoV-2 can disrupt the blood–brain barrier via direct or indirect pathways entailing mast cell and microglial activation [31]. In vitro experiments demonstrated transcellular transport of the SARS Co2 virus across the blood–brain barrier [32]. Furthermore, chronic cytokinemia with proinflammatory cytokines, such as IL-1β, interleukin-2, or IL-6, and also antibodies like those against interferon-α, interferon-λ, C-C motif chemokine ligand 26, or CXC motif chemokine ligand 12, may lead to changes in the blood–brain barrier, and thus, cause neurotoxicity [33]. Such increased blood–brain barrier permeability may allow autoantibodies to enter and accumulate in the brain, thus impairing various brain functions. Multicenter study evidence [34] has shown that the blood–CSF barrier is also disturbed. However, while no intrathecal inflammation is detected in most cases of COVID-19, cerebrospinal endotheliopathy is present [34].

### 4.5. Bystander Activation, Epitope Spreading, and Autoantibodies

Microglia could lead to a stronger release of cytokines, triggering a local proinflammatory state in the brain, which would, in turn, release additional self-antigens such as myelin in antigen-presenting cells, thus causing even more brain tissue damage (termed bystander activation). The continued presentation of the self-antigen by antigen-presenting cells triggers T-cell activation. These T-cell responses spread to self-antigens, thus instigating autoimmunity (termed epitope spreading) and autoantibody production.

## 5. Neural Autoantibodies in COVID-19 with Psychiatric Manifestation

A recent study by Franke [1] investigated neural autoantibodies in 11 neuropsychiatric patients (Table 1). They reported on three patients exhibiting delirium, together with other symptoms such as myoclonus or nystagmus, which, in turn, were associated with neural autoantibodies in the serum (N-methyl-D-aspartate (NMDAR) and myelin). These findings were not confirmed in the cerebrospinal fluid (CSF), however. Overall, neural autoantibodies were detected in 4 of the 11 (36%) COVID-19 patients investigated in their CSF analysis. Indirect immunohistochemical studies on mouse brain sections also revealed the nonspecific binding of neural autoantibodies to hippocampal, cerebellar, olfactory neuropil, or brainstem neuropil tissue and to unknown brain antigens [1]. Another interesting single report [4] described a man (whose anti-neural autoantibodies were assessed) with subacute psychosis also suffering from delusions and agitation. The authors detected no specific neural autoantibodies, but his CSF IgG showed immunostaining of the olfactory bulb, cortex, thalamus, and hippocampus. The patient’s CSF revealed enriched autoantigen multiple C2 and transmembrane domain-containing 1 (MCTP1), an essential nervous system endosome protein involved in neurotransmitter release. However, the MCTP1 autoantigen was not overexpressed in cell-based assays, but it was significantly more enriched than in the control CSF samples and sera. These case series indicate that neural autoantibodies are more prevalent in patients with psychiatric symptoms and COVID-19 than in the normal population, although the evidence is not yet conclusive. Another important study [3] (Table 1) in 169 COVID-19 patients demonstrated dysregulated circulating autoantibodies for brain antigens in the blood compared to 77 control subjects. The levels of immunoglobulin A (IgA) autoantibodies for the acetylcholine receptor, dopamine 2 (D2) receptor, and myelin oligodendrocytic protein (MOG) were elevated in COVID-19 patients regardless of their disease activity [3]. Furthermore, antibodies against NMDAR, brain-derived neurotrophic factor (BDNF), glutamic acid decarboxylase (GAD65), and the dopamine 1 (D1) receptor were increased in patients with severe COVID-19 and in those requiring oxygen [3]. These findings suggest dysregulated autoantibody levels in patients with COVID-19 compared to the controls, with their prevalence often higher in severe disease states of COVID-19, supporting the triggering of autoimmunity in SARS-CoV-2 infection. Another study [6] demonstrated that autoantibodies against various neuronal antigens, such as anti-myelin-associated glycoprotein, were detected in 9.6% of COVID-19 samples but not in the controls. However, autoantibodies against neuroendocrine antigens, such as antipituitary antibodies and antihypothalamic antibodies, were also observed in COVID-19 patients [35]. Furthermore, it is possible that the production of autoantibodies against various CNS antigens is due to a leaky blood–brain barrier followed by the production of autoantibodies against CNS antigens. In addition, brain injury in COVID-19 is known to be associated with inflammation [6], which could facilitate immune responses involving autoantibodies being produced against various antigens. The wide spectrum of autoantibodies against different brain antigens may also result from the fact that a very widespread immune response also affects the brain.

## 6. Neural Autoantibodies in Post-COVID-19 Syndrome with a Psychiatric Manifestation

Another recent investigation by [5] detected anti-neuronal antibodies in 52% of 50 patients. These neuronal antibodies were directed against antigens such as Yo, Ma2/Ta, GAD65, NMDAR, and a variety of undetermined epitopes of antigens on brain sections. Another interesting finding from their study was a strong correlation between dysfunctional cognitive testing based on the Montreal Cognitive Assessment (MoCA) test and the presence of antineuronal antibodies [5]. These results suggest that neural autoantibodies in PCS appear to be associated with a phenotype characterized by cognitive impairment. Another recent study examined the frequency of autoantibodies by applying proteome-wide autoantibody detection technology. A total of 121 patients suffering from prolonged COVID-19 and patients with previous COVID-19 and total recovery, and 57 pre-COVID-19 control subjects, were studied in this investigation [2] (Table 1). Activating-Rho GTPase-Activating Protein (ARHGAP31) was the major autoantigen identified in 22% of subjects with a prior SARS-CoV-2 infection and achieved enrichment with a 6-fold increase compared with the pre-COVID-19 controls [2]. Interestingly, a region in the SARS-CoV-2 open reading frames 1a polyprotein showed significant physicochemical similarity to the autoreactive fragment of ARHGAP31, supporting the interpretation that enriched human peptides in post-COVID-19 samples are driven by SARS-CoV-2 antibodies [2]. Interestingly, in long COVID-19 patients, 17 of 20 enriched proteins were detected at a fivefold increase, but not in pre-COVID-19 patients [2]. However, this method failed to reveal an autoantibody pattern that distinguished between patients with long COVID-19 and those who have fully recovered from SARS-CoV-2 infection [2]. Furthermore, a recent study showed that anti-GAD65 antibodies were also more elevated in patients with long COVID-19 disease. Interestingly, higher titers of GAD65 antibodies were correlated with lower levels of attentional and executive function and working memory [36]. More research is needed to identify a pattern of neural autoantibodies or specific autoantibodies clustered in long-COVID patients with psychiatric symptoms.

## 7. Potential Brain Damage in Autoantibody-Associated COVID-19 and Post-COVID-19 Syndrome with Neuropsychiatric Symptoms

**Figure 1:**
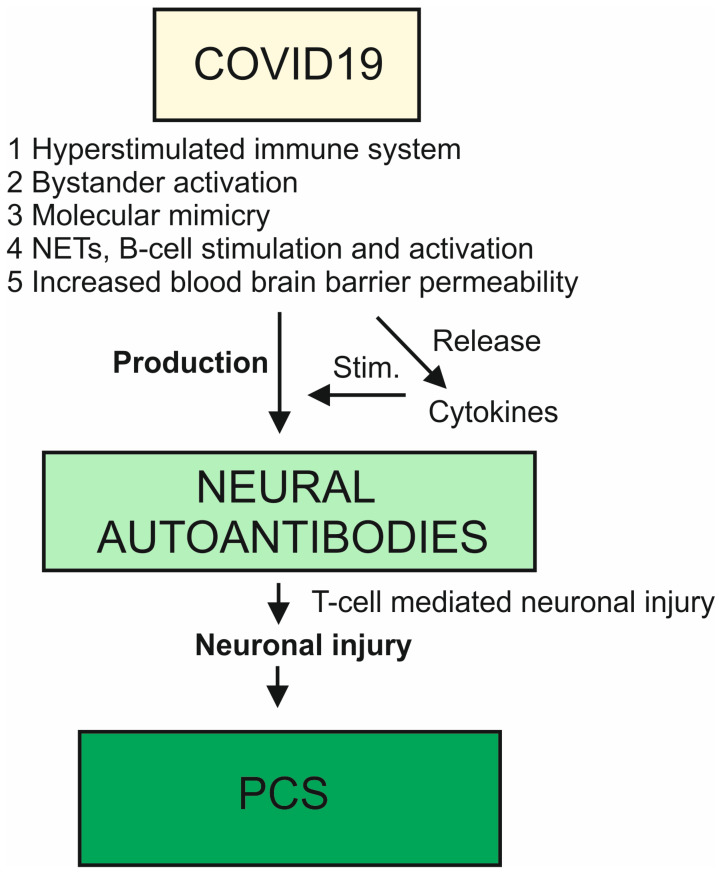
Schematic representation of neural autoantibodies in the context of COVID-19 and post-COVID-19 syndrome with neuropsychiatric symptoms.

There is recent evidence from a 105-patient cohort from Geneva that in a long-term 6–9-month interval after a COVID-19 infection, COVID-19 infection severity affected the development of neuropsychiatric symptoms [37]. Recognition was more impaired in patients with moderately severe COVID-19 disease than in patients with mildly severe COVID-19 disease 6–9 months after infection [37]. These findings indicate that in the long term, cognitive impairment is more pronounced when COVID-19 infection is initially moderate to severe. Functional-connectivity analysis of the Geneva cohort found a pattern suggesting decreased functional connectivity between the cerebellum and subcortical and cortical networks in these patients, with deficits in recognizing fear and irritation in patients with mild and moderate severity [37]. This is evidence of functional-connectivity anomalies in patients with psychiatric symptoms after COVID-19. In addition to changes in functional-connectivity networks between the cerebellum and cortical structures, changes in blood levels in the biomarkers of brain injury are also likely. A biomarker study with sera from 175 patients showed that markers of axonal and astroglial neuronal brain injury are elevated for up to 4 months, suggesting relevant brain injury after COVID-19 [6]. It is very likely that autoantibody-associated PCS involving psychiatric symptoms is also associated with relevant brain damage. In a recently published study of 29 PCS patients who complained of memory and concentration problems, the patients’ performance was surprisingly similar on a battery of tests (NIHTB-CB = National Institute of Health Toolbox Cognition Battery) for mental performance and emotions, revealing increased activity in the right superior frontal gyrus and decreased activation in the default network [38]. However, these patients also obtained higher scores for negative affect and perceived stress and lower scores for well-being in the NIHTB-EB (NIHTB-EB = National Institute of Health Toolbox Emotion Battery) [38]. We therefore suspect that compensatory and reorganizational activity is already occurring at the brain level to compensate for cognitive deficits or emotional stress. Nonetheless, no study to date has shown a correlation between brain injury in psychiatric patients with PCS and associated neural autoantibodies. More research investigating large cohorts is thus needed to discover any specific pattern of neuronal cell damage or a specific pattern in imaging with limbic system involvement in these patients. The limbic system revealing involvement of the parahippocampal gyrus and orbitofrontal cortex has been reported in patients with COVID-19, as verified in a large biobank cohort in the United Kingdom [39], probably via an inflammation route through the olfactory system. The authors of a recent review postulated that neurotoxicity can be induced via chronic cytokinemia and transient opening of the blood–brain barrier [33], resulting in brain injury. The entry of autoantibodies via an open blood–brain barrier could trigger additional brain damage (see Figure 1). These authors also suggest that the development of autoantibodies in particular may affect various other regulatory processes in the brain such as repair mechanisms, microglial function, or neurogenesis [33], or bioelectrical activity, resulting in neuropsychiatric symptoms [40], thus causing permanent damage to key brain functions. There are general indications suggesting the presence of autoimmunity in COVID-19 infection as well as possible PCS (Figure 1); this is supported by the presence of autoantibodies against specific neural antigens or even unknown neural antigens (Figure 1), but also by the presence of autoimmune-related cytokines (Figure 1) or cellular activity as in autoimmune diseases [41].

After COVID-19 infection, mechanisms such as the blood–brain barrier’s opening and the rise in cytokines trigger the production of neural autoantibodies, which may be associated with increased neuronal brain damage. Through a T-cell mechanism, autoantibodies may result in brain damage, which, in turn, may cause post-COVID-19 syndrome (PCS). Abbreviations: Stim. = stimulation, NETs = neutrophil extracellular traps.

## 8. Therapeutic and Biomarker-Supported Approach

Taken together, the evidence so far suggests that there is a growing number of neuropsychiatric patients with PCS in whom neural autoantibodies could be identified provided they undergo routine diagnostics, as this is a significant phenomenon with potential therapeutic consequences. In addition to a broad panel of neural autoantibodies against both intracellular (Yo, Ma2/Ta2, GAD65, ARHGAP26/31) and membrane surface structures (MOG, myelin, NMDAR, D1/D2), which should be sought in cerebrospinal fluid and blood, the determination of cytokines and other inflammation markers, as well as neuronal brain damage (Nfl, GFAP, tau proteins), is useful for finding a proxy for potential brain damage. In this context, it is especially important to seek autoantibodies against intracellular antigens, since a large cohort study (with 15,390 patients) showed that before and during the pandemic, the frequency of autoantibodies against neuronal antigens (3.2% vs. 3.5%) and glial antigens (6.1% vs. 5.2%) did not change. In contrast, however, there was a dramatic increase in autoantibodies for intracellular antigens during the pandemic (2.8%. vs. 3.9%) [42]. Therefore, it is significant that investigators are looking for autoantibodies against intracellular antigens. Here, autoantibodies against intracellular antigens such as Hu and GFAP are probably especially relevant, as the study by [42] showed. Several theoretical lines of evidence indicate that autoimmunity may rise after COVID-19 and in PCS. Nevertheless, as autoantibodies for membrane surfaces are not elevated in patients [42], autoimmune processes involving T-cells in particular may be more strongly activated by COVID-19 (Figure 1). This aspect should be investigated in future studies. According to Arino’s article [42], the COVID-19 pandemic has not led to a substantial increase in autoimmune encephalitis associated with membrane surface autoantibodies. They report that the increase in Hu and GFAP antibodies is attributable to a rise in the detection of corresponding diseases associated with these autoantibodies. However, it is essential to note that not all psychiatric patients with COVID-19 or PCS and neural autoantibodies have had autoimmune encephalitis. This statement can therefore not be generalized, but rather, should be considered directional for autoimmune encephalitis due to their large patient cohort.

With respect to a diagnostic pathway for patients with psychiatric symptoms persisting after COVID-19 infection, it is imperative that structural brain exams such as cMRI or the assessment of glucose metabolic activity via 18F FDG PET be conducted to detect morphometric or metabolic abnormalities potentially leading to the need for therapy. When seeking autoantibodies in conjunction with psychiatric symptoms, we recommend following the guidelines for autoimmune encephalitis [43,44]. If those criteria are not met, other criteria for autoimmune genesis are accessible [13] to provide a rationale for employing off-label immunotherapy as individualized treatment. We suggest a staged approach to immunotherapy analogous to the criteria for autoimmune encephalitis [43,44]. Steroids or intravenous immunoglobulins should be considered as first-line therapy, and plasmapheresis should be considered in severe cases. As second-line therapy, steroid-sparing agents such as methotrexate or azathioprine can be applied. Monoclonal antibody therapy as additional therapy-escalation is a potential ultima ratio. Such therapy requires interdisciplinary cooperation with colleagues from immunology, neurology, psychiatry, psychotherapy, cardiology and pneumonology, for example [45]. Due to the frequently acute onset of symptoms, and their occurrence during the disease course, close cooperation with admissions and intensive care units is essential. Because of their high prevalence and increased comorbidity risk, autoantibodies other than neural autoantibodies are relevant, such as antinuclear autoantibodies. A recent study [46] showed that vaccination in healthcare workers also led to a significant increase in de novo autoantibodies against nuclear antigens. Such study evidence makes it clear that an interdisciplinary approach is best, and that in patients presenting neuropsychiatric or purely psychiatric symptoms and suspected PCS, it is advisable to assess both neural autoantibodies and antinuclear antibodies.

## 9. Synopsis

On the one hand, these pilot studies indicate that neural autoantibodies (52–100%) [1,5] appear to be much more frequent in COVID-19 and PCS patients with psychiatric or neuropsychiatric symptoms than in healthy controls (15%) or psychiatric patients (10–23%), as shown in 7000 patients with 49 autoantibodies [47]. Moreover, autoantibodies against unknown target antigens have been detected even more frequently in both COVID-19 and PCS patients, supporting the hypothesis that SARS-CoV-2 virus infection is a relevant trigger for autoimmunity and autoantibody production, mainly via the aforementioned mechanisms. New target antigens such as ARHGAP31 are also emerging that are potentially related to COVID-19-associated autoimmunity. More research with large cohorts is urgently needed to develop strategies that facilitate the early detection and treatment of psychiatric patients with COVID-19 or PCS who may possess neural autoantibodies. COVID-19 could serve as a model system showing us how autoimmunity can be triggered by an antecedent viral infection, as the COVID-19 pandemic is widespread and ubiquitous enough that studies can be conducted with sufficient statistical power, and SARS-CoV-2 infection can be severe enough to trigger such autoantibody production as a facet of autoimmunity following viral infections. Other factors should be considered together, i.e., other non-neural autoantibodies against chemokines [48] or other functional autoantibodies affecting the immune system’s capacity and clinical course [49]. In sum, it is essential that we conduct more intensive research to improve overall care for neuropsychiatric patients presenting neural autoantibodies in a COVID-19 context, but we also need to consider COVID-19 as an exemplary system for studying psychiatric disorders associated with COVID-19.

## 10. Summary

1. Neural autoantibodies are detected more frequently in patients presenting neuropsychiatric symptoms, COVID-19, and PCS than in controls.
2. The spectrum of psychiatric symptoms in patients with COVID-19 and PCS associated with neural autoantibodies ranges from cognitive impairment to psychosis.
3. The autoantibodies identified in neuropsychiatric patients with COVID-19 and PCS are ARHGAP31, GAD65, acethylcholine receptor, D1/2, MOG, NMDAR, MCTP1, Yo, myelin, and Ma/Ta2.
4. The pathomechanisms of possible COVID-19 and PCS disorders accompanied by psychiatric symptoms and evidence of neural autoantibodies include molecular mimicry, a hyperstimulated immune system, NETs, altered blood–brain barrier permeability, and bystander activation.
5. There is indirect evidence from biomarker studies that COVID-19 and PCS with autoantibodies can lead to brain damage.
6. Therapeutic approaches should follow the guidelines for known autoimmune conditions, such as autoimmune encephalitis, to provide individualized treatment.

## Figures and Tables

**Table 1:** Neural autoantibodies in neuropsychiatric patients with COVID-19 or post-COVID-19 syndrome.

DISEASE	COVID-19	COVID-19	COVID-19	COVID-19	COVID-19	PCS
**PATIENT** **NUMBERS**	N = 64 with prior COVID-19 vs. N = 57 pre-COVID-19 controls	N = 121 with long COVID-19 vs. N = 64 with prior COVID-19 and full recovery	N = 169 with COVID-19 vs. N = 77 controls	N = 1 with COVID-19	N = 11 with COVID-19	N = 50 with PCS
**NEURAL** **AUTOANTIBODY SPECTRUM**	Most prominent autoreactivity: ARHGAP31	Most prominent autoreactivity: ARHGAP31	GAD65, acetylcholine receptor, D1/D2 receptor, Myelin Basic Protein, MOG, NMDAR	MCTP1	NMDAR, Yo, myelin, unknown target antigen	Yo; Ma/Ta2, GAD65, NMDAR, undetermined epitopes of antigens on brain sections
**CSF**	−	−	−	+	+	
**BLOOD**	+	+	+	+	+	
**RESULTS**	Autoreactive signature in patients with prior COVID-19 vs. pre-COVID-19 controls	No autoreactive signature in patients with prior COVID-19 vs. pre-COVID-19 controls detected	Levels of IgA autoantibodies against acetylcholine receptors, D2 receptors, and myelin basic protein elevated in COVID-19 patients vs. controls regardless of the disease severity. Levels of IgG autoantibodies against NMDAR, GAD65, D1R, and MOG elevated in patients with severe form of COVID-19 and the need for oxygen. Levels of NMDAR IgA lower in COVID-19 patients than in controls	Enriched MCTP1 significantly higher than in combined 3408 healthy CSF and sera and 808 negative controls	A total of 4 in 11 (36%) COVID-19 patients positive for neural autoantibodies in CSF All patients had anti-neural autoantibodies, but some were unspecific	Strong correlation between dysfunctional cognitive performance on the MoCA test and presence of antineuronal antibodies detected in 52% of 50 patients
**REFERENCE**	[2]	[2]	[3]	[4]	[1]	[5]

Abbreviations: ARHGAP31 = Activating-Rho GTPase-Activating Protein, COVID-19 = coronavirus disease 2019, CSF = cerebrospinal fluid, D1/D2 receptor = dopamine 1 and dopamine 2 receptor, GAD65 = glutamic acid decarboxylase 65, MoCA = Montreal Cognitive Assessment, MOG = myelin oligodendrocytic protein, N = number, NMDAR = N-methyl-D-asparate-receptor.

## Data Availability

The data generated for this article are available from the author with undue reservation.
